# Effects of Reduced Summer Precipitation on Productivity and Forage Quality of Floodplain Meadows at the Elbe and the Rhine River

**DOI:** 10.1371/journal.pone.0124140

**Published:** 2015-05-07

**Authors:** Kristin Ludewig, Tobias W. Donath, Bianka Zelle, R. Lutz Eckstein, Eva Mosner, Annette Otte, Kai Jensen

**Affiliations:** 1 Biocenter Klein Flottbek, University of Hamburg, Hamburg, Germany; 2 Institute of Landscape Ecology and Resource Management, Research Centre for Biosystems, Land Use and Nutrition (IFZ), Justus Liebig University Giessen, Giessen, Germany; 3 Bundesanstalt für Gewässerkunde (BfG), Koblenz, Germany; Fudan University, CHINA

## Abstract

**Background:**

Floodplain meadows along rivers are semi-natural habitats and depend on regular land use. When used non-intensively, they offer suitable habitats for many plant species including rare ones. Floodplains are hydrologically dynamic ecosystems with both periods of flooding and of dry conditions. In German floodplains, dry periods may increase due to reduced summer precipitation as projected by climate change scenarios. Against this background, the question arises, how the forage quantity and quality of these meadows might change in future.

**Methods:**

We report results of two field trials that investigated effects of experimentally reduced summer precipitation on hay quantity and quality of floodplain meadows at the Rhine River (2011-2012) and at two Elbe tributaries (2009-2011). We measured annual yield, the amount of hay biomass, and contents of crude protein, crude fibre, energy, fructan, nitrogen, phosphorus, and potassium.

**Results:**

The annual yield decreased under precipitation reduction at the Rhine River. This was due to reduced productivity in the second cut hay at the Rhine River in which, interestingly, the contents of nitrogen and crude protein increased. The first cut at the Rhine River was unaffected by the treatments. At the Elbe tributaries, the annual yield and the hay quantity and quality of both cuts were only marginally affected by the treatments.

**Conclusion:**

We conclude that the yield of floodplain meadows may become less reliable in future since the annual yield decreased under precipitation reduction at the Rhine River. However, the first and agriculturally more important cut was almost unaffected by the precipitation reduction, which is probably due to sufficient soil moisture from winter/spring. As long as future water levels of the rivers will not decrease during spring, at least the use of the hay from the first cut of floodplain meadows appears reliable under climate change.

## Introduction

European semi-natural habitats such as agriculturally unimproved grasslands make a large contribution to the species diversity of landscapes [1, 2]. This is not only due to the high plant species richness, which is characteristic for semi-natural grasslands, but also due to large numbers of animal species (e.g. insects and birds) for which grasslands offer suitable habitats (e.g. [3]). Floodplain grasslands along large lowland rivers are hotspots of biodiversity and the outcome of typical flooding regimes and long lasting land use practices with moderate intensity [4]. They harbour many rare [5, 6], typical river corridor plants such as *Cnidium dubium*, *Thalictrum flavum* or *Viola elatior* [7]. Due to the impact of flooding events in combination with dry conditions over the summer, floodplains are highly dynamic and variable environments [8]. Floodplains are mainly used as grasslands for grazing (pastures) and mowing (meadows) to gain fodder for domestic livestock. Mowing once or twice annually without fertilizer application is recommended for facilitating a high species richness [4].

As other semi-natural grasslands in Europe, floodplain meadows strongly declined over the last centuries. The main causes for loss of species-rich floodplain meadows are the reduction of the dynamic hydrologic conditions due to river regulations [9] or river training [10], and land use changes as intensification (e.g. [11]) or abandonment (e.g. [12]). Consequently, these meadows are of high conservation value and certain types of meadows found in European floodplains are protected by the Habitats Directive (92/43/EEC, habitat type 6440: Alluvial meadows of river valleys of the *Cnidion dubii*; 6510: Lowland hay meadows) and subject to various restoration measures [13–15].

It is an ongoing nature conservation concern how the biomass of species-rich non-intensively managed meadows can be incorporated into agricultural land use systems [16–18] and animal nutrition. Undoubtedly, it is preferable to integrate these meadows into farming systems instead of just managing them in the framework of nature conservation schemes. In future, however, the productivity of semi-natural grasslands may be affected by increased rainfall variability due to climate change.

Precipitation is one of the most influential abiotic factors for plant productivity [19]. Changes in precipitation patterns are projected to occur in the course of climate change [20]. For Germany, regional climate change projections indicate higher temperatures and an increasing risk for summer droughts for the late 21st century due to less summer precipitation in relation to the reference period 1961–1990 [21, 22]. Accordingly, river discharges during summers are projected to decrease, e.g. at the large rivers Rhine [22] and Elbe [23]. This, in turn, could lower the water table in the adjacent floodplains with negative effects on the soil water potential. In combination with increased transpiration at higher temperatures, these changes could induce drought stress in plants of floodplain meadows [24]. Plant responses to drought stress are manifold, including decreased cell elongation and reduced photosynthesis; the responses generally lead to reduced plant growth and delayed plant development [25].

Nitrogen fertilization generally affects the vegetation of semi-natural grasslands: besides increased productivity, shifts in species compositions were observed [26, 27]. With respect to forage quality, mostly increases [28, 27] but also decreases in nitrogen contents [26] were recorded after nitrogen addition. How nitrogen addition affects the vegetation of floodplain meadows is less known.

In recent years, some climate change experiments were conducted in grassland ecosystems, e.g. in a semi-arid steppe [29], in mesic tallgrass prairies [30], and in temperate grasslands [31–34]. Only one of these studies focused on how forage quality—the ecosystem service relevant to farmers—might change in the future using the example of nitrogen and protein content of the biomass [34]. Still, additional variables relevant for farmers such as crude fibre, crude protein, energy content or fructan have not been analysed in this context. Generally, data on the forage quality of non-intensively used semi-natural grasslands are scarce in the international literature (but see [17, 18, 35]). Also in the UK, data for biomass from species-rich semi-natural grasslands are lacking [36] and it seems that there is still a current need to examine this topic. Generally, high values of crude protein, digestible energy and contents of nitrogen (N), phosphorus (P), and potassium (K) indicate high forage quality of hay while high amounts of crude fibre and fructan impair forage quality [37].

To clarify the effects of changes in summer precipitation on the quantity and quality of the biomass of European floodplain grasslands, we conducted field experiments in which we reduced the amount of precipitation with rainout shelters. We harvested biomass, analysed its amount and the above mentioned parameters. Further, we calculated the annual yield as the product of biomass amount and energy content. The data we present here originate from two independent precipitation experiments, conducted at the rivers Rhine and at two Elbe tributaries.

We aimed at answering the following research questions: Does reduced summer precipitation affect the quantity of hay, its quality in the first and second cut, and the overall annual energy yield of floodplain meadows?

## Materials and Methods

### Experiments at the Elbe and the Rhine River

We summarise results of two precipitation reduction experiments in floodplain meadows at the Rhine River and at two Elbe River tributaries originating from two independent studies. Because the two studies were planned and conducted independently from each other, different experimental designs were employed. However, as the overall aims and the used rainout shelters were identical, we present the results in this integrating paper.

### Permissions

The permits for the field experiments were provided by the Biosphere Reserves Elbe River of Lower Saxony for the experiment at the Sude, from the Biosphere Reserves Elbe River of Saxony-Anhalt as well as from the Nature Conservation Authority from Saxony-Anhalt for the Havel. Permits for the experiment at the Rhine were provided from the city of Riedstadt, the regional council Darmstadt and the forestry administration of Hesse.

### Study areas and study sites

The studies were conducted on floodplain meadows belonging to the functional floodplain (not disconnected from the river and thus inundated in times of high water) at the Rhine River and at two Elbe River tributaries, the Havel and the Sude River. All studied floodplains have in common that the climatic and hydrological conditions result in highly variable soil water potentials: while winter, spring and early summer may bring floods, the summer periods are notably dry (especially the continental Havel site in the Elbe region and the Rhine site). Along with the strong seasonal and inter-annual fluctuations of the water level of the rivers, the groundwater tables also strongly fluctuate [38, 39].

The study area at the Rhine River is situated in the Hessian part of the Holocene floodplain of the northern Upper River Rhine near Riedstadt, about 30 km southwest of Frankfurt, Germany (N 49°49′, E 8°26′). The climate in this area is relatively dry and warm with a mean annual precipitation of 602 mm (1961–1990 Riedstadt-Erfelden, DWD 2013) and a mean annual temperature of 9.7°C (1961–1990 Frankfurt Main airport, DWD 2013). The soils can be classified as calcic Fluvisols [40]. The vegetation of the study site itself is relatively species poor because it was an arable field until 1983 (for details on the history of the site see [41, 42]). Since 1983, the meadows are mown up to two times annually. Two sites differing in elevation were chosen as study sites (a higher/dryer site and a lower/wetter site). The species composition of the study meadow is dominated by *Festuca arundinacea*. Further frequently occurring species are *Dactylis glomerata* and *Leucanthemum vulgare* on the higher site and *Potentilla reptans* and *Symphytum officinale* on the lower site. The nomenclature of plant species follows Wisskirchen and Haeupler [43].

The meadows at Elbe tributaries are located at the Sude River (near Sückau in Lower Saxony, N53°19′ E010°57′) and the Havel River (near Kuhlhausen in Saxony-Anhalt, N52°47′ E012°11′). The site at the Sude River experiences rather oceanic climatic conditions with a mean annual precipitation of 663 mm and a mean annual temperature of 8.3°C (1961–1990; data from the nearest weather station in Boizenburg; DWD 2013). The site at the Havel River has on the contrary relatively continental climatic conditions with a mean annual precipitation of 503 mm (1961–1990; data from the nearest weather station in Havelberg; DWD 2013) and a mean annual temperature of 9.1°C (1976–2009; data from the nearest weather station measuring temperature in Seehausen; DWD 2013). Both sites are regularly flooded by either the Sude or the Havel River, which are first order tributaries of the Elbe River. The soils of both sites are gleyic Fluvisols, which consist mainly of loamy material over sandy sediments. The active floodplains along the Sude and Havel are typically used as grasslands and the two meadows are mown twice annually. Both study sites contain *Cnidium*-floodplain meadow vegetation with characteristic river corridor plants (according to [7]), such as *Cnidium dubium*, *Stellaria palustris* and *Carex vulpina*, and more frequently occurring species, such as *Alopecurus pratensis*, *Deschampsia cespitosa*, *Potentilla anserina*, *Potentilla reptans*, and *Ranunculus repens*.

### The experiment at the Rhine River

**Fig 1:**
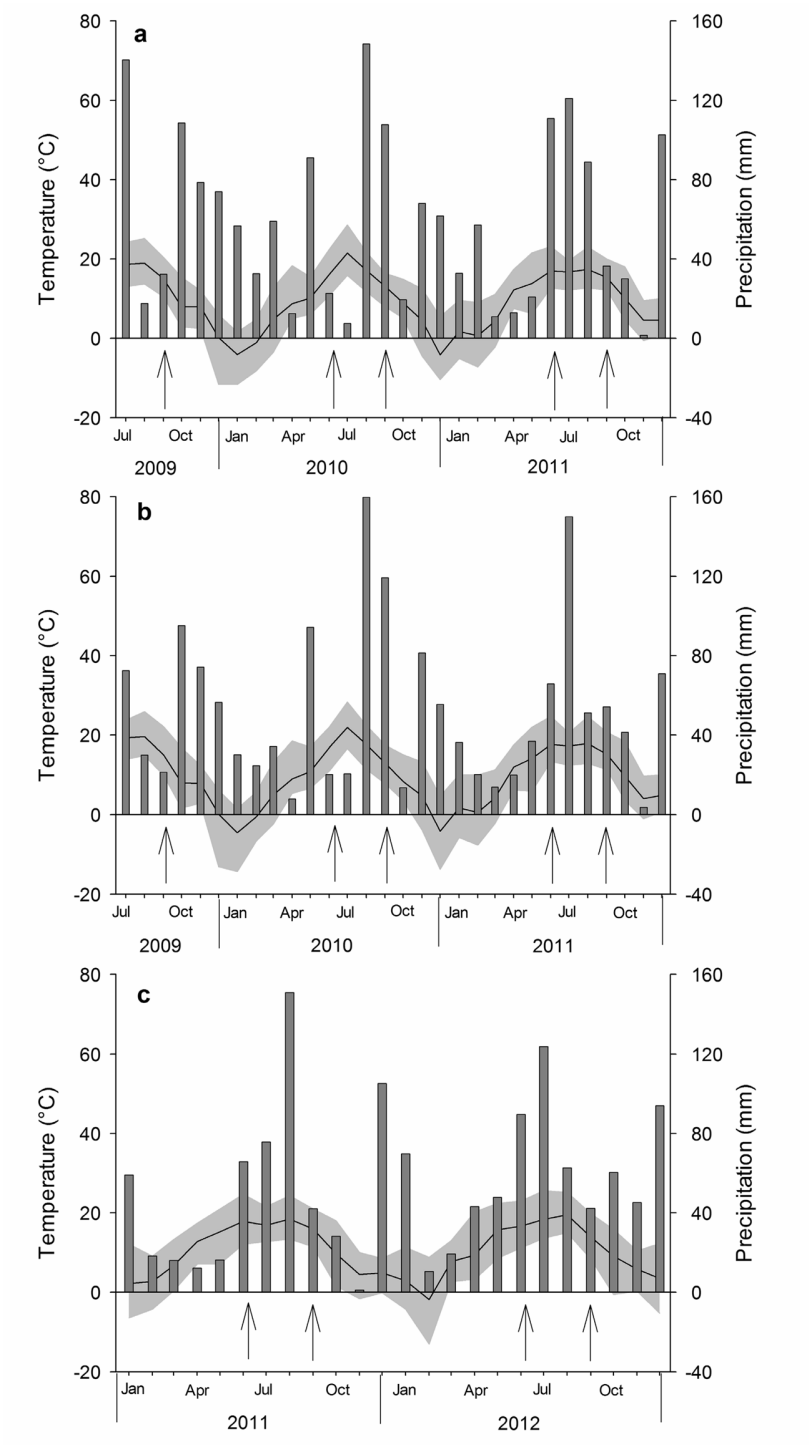
Weather conditions at the Elbe tributaries Sude (a) and Havel (b) during the study years 2009 to 2011 and at the Rhine (c) during the study years 2011 and 2012 (data provided by the DWD 2013). The black line with grey shade represents daily average, minimal and maximal monthly temperatures. The grey bars are monthly sums of precipitation. Arrows indicate the dates of biomass sampling.

The experiment at two sites at the Rhine River was conducted in the vegetation periods 2011 and 2012. The rainout shelters measured 3 m x 3 m and were built according to Yahdjian & Sala [44] using acrylic glass pipes as flumes. To minimize edge effects, the investigated plots beneath the rainout shelters were adjusted to 4 m^2^. The two studied experimental factors were elevation above base flow (high: 320 cm above base flow; low: 240 cm above base flow) and precipitation reduction (-50%, -25%, no reduction). Two types of controls were used: 1) control plots with rainout-shelters where the acrylic glass pipes were turned upside down (to test the rainout-shelter effect without rain reduction) and 2) control plots without rainout shelters (see Table 1). Precipitation reduction was conducted from March to October. The experiment was run with three replicates. The weather conditions of the study years are shown in Fig 1c.

**Table 1:** Overview of experimental treatments of the precipitation reduction experiments at the Elbe tributaries and the Rhine: -50% = 50% precipitation reduction; -25% = 25% precipitation reduction; +N = fertilization with N; control = controls without rainout shelters; control+shelter = controls with rainout shelters.

	-50%	-25%	+N /-25%	+N	control	control+shelter
Elbe tributaries		2009–2011	2009–2011	2009–2011	2009–2011	
Rhine	2011–2012	2011–2012			2011–2012	2011–2012

### The experiment at the Elbe River tributaries

In the vegetation periods 2009 to 2011, we conducted a field experiment at the two Elbe River sites. In a two-factorial design, we manipulated summer precipitation, which was reduced by approx. 25% with rainout shelters, and N deposition, which was imitated by fertilization with ammonium-nitrate (35 kg N ha^-1^ a^-1^). Precipitation reduction and fertilization treatments were conducted from May to October (in 2009 from July to October). Fertilizer was applied at seven dates evenly distributed over this period. As in the Rhine experiments, the rainout shelters measured 3 m x 3 m and were built according to Yahdjian & Sala [44], but using UV permeable greenhouse plastic as flumes. To minimize edge effects, study plots covered only the inner approx. 4 m^2^ of the rainout shelter. Four treatments were implemented (see Table 1): 1.) precipitation reduction and fertilization (-25%/+N), 2.) only fertilization (+N), 3.) only precipitation reduction (-25%), and 4.) controls without treatments (controls). The experiment was run with seven replicates. The weather conditions of the study years are shown in Fig 1a and 1b.

### Response variables

As response variables we measured the amount of biomass (g m^-2^), its contents of crude fibre (XF, % dry weight; dw), crude protein (XP, % dw), the elements N (mg g^-1^ dw), P (mg g^-1^ dw), and K (mg g^-1^ dw) and fructan (% dw). Fructans are storage carbohydrates of many grass species [45]. High contents of fructan in the forage can be unhealthy for horses and ponies [46]. Energy content of the biomass was assessed as digestible energy (DE), net energy for lactation (NEL) and metabolisable energy (ME; all in MJ kg^-1^ dw). While the latter two are applicable in case of ruminants, DE is an estimate relevant for horse fodder. As these energy values are interrelated and the hay of semi-natural meadows is per se preferably used for horses in the study regions, we focus on DE in this study, but as an overview, we present the ME- and NEL-values in Tables 2 and 3.

**Table 2:** Forage quality parameters of differently treated meadow plots (control, control +shelter), -25% precipitation, and -50% precipitation) on the moist and dry meadow site in the floodplain at the Rhine River.

	Moist site								Dry site												Significance
	control		control+shelter		-25%		-50%		control			control+shelter			-25%			-50%			between sites
	*x*	SE	*x*	SE	*x*	SE	*x*	SE	*x*		SE	*x*		SE	*x*		SE	*x*		SE	*p*
**June 2011**																					
Biomass	270.7	22.6	291.6	31.6	233.9	43.0	261.3	54.0	140.5		5.2	147.1		10.0	125.1		6.3	124.0		4.6	0.0003
XF	30.8	0.6	32.4	0.4	33.2	1.3	32.3	<0.1	36.0		0.9	37.1		1.1	36.4		0.3	36.5		0.6	0.0002
XP	9.9	0.5	10.0	1.0	9.1	0.8	8.0	0.4	12.0		0.6	11.4		0.5	11.9		0.7	10.7		1.1	0.0002
DE	8.3	0.1	7.8	<0.1	7.6	0.3	8.0	0.1	6.9		0.2	6.7		0.3	6.8		0.1	6.7		0.2	0.0002
ME	8.4	0.1	7.9	0.2	7.8	0.1	8.2	0.2	7.0		0.2	6.9		0.3	6.8		0.3	6.8		0.5	0.0002
NEL	4.9	0.1	4.5	0.1	4.4	0.1	4.7	0.1	4.0		0.1	3.9		0.2	3.8		0.2	3.8		0.3	0.0002
Fructan	3.2	0.4	2.7	0.3	2.9	0.5	4.3	0.2	0.4		0.1	0.6		0.2	1.4		0.1	1.9		0.3	0.0002
N	12.6	1.0	13.7	1.0	13.2	1.0	11.2	0.7	17.0		0.6	15.6		0.4	16.7		0.5	15.5		1.1	0.0002
P	2.6	0.1	2.6	0.1	2.7	0.1	2.6	0.1	3.8		0.3	3.6		0.1	3.6		0.1	3.1		<0.1	0.0002
K	16.3	0.9	16.0	0.6	16.1	1.7	15.5	0.1	16.8		1.0	17.0		1.1	16.8		0.7	15.7		0.5	0.6897
**June 2012**																					
Biomass	340.1	33.4	364.2	32.5	324.2	74.6	204.3	54.5	180.1		3.3	215.7		2.1	168.3		17.6	165.9		13.5	0.0003
XF	34.5	0.4	35.6	0.5	35.3	1.1	34.1	0.3	37.1		0.4	39.4		0.4	37.6		0.6	40.4		0.6	0.0002
XP	8.5	0.5	6.9	0.1	7.5	0.7	7.9	0.5	6.2		0.3	5.6		0.2	6.1		0.1	4.8		0.3	0.0004
DE	7.5	0.1	7.3	0.2	7.5	0.2	7.8	0.1	7.2		0.1	6.7		0.1	7.2		0.1	6.6		0.1	0.0003
ME	7.5	0.2	7.6	0.1	7.6	0.2	7.9	0.1	7.6		0.1	7.2		0.1	7.6		0.2	7.1		0.1	0.2144
NEL	4.3	0.1	4.3	0.1	4.3	0.2	4.5	0.1	4.3		0.1	4.1		0.1	4.3		0.1	3.9		0.1	0.1854
Fructan	2.6	0.7	3.0	0.7	4.0	0.5	4.3	0.5	1.8		0.1	0.8		0.2	1.8		0.4	1.4		0.5	0.0002
N	12.9	0.9	11.2	0.4	11.2	1.1	12.4	0.7	10.6		0.4	9.7		<0.1	10.3		0.1	9.0		0.2	0.0021
P	2.6	0.1	2.6	0.1	2.7	0.1	2.8	0.2	4.1	a	0.3	3.8	ab	0.1	3.9	a	0.1	3.0	b	0.1	0.0002
K	16.1	0.4	16.1	0.5	14.5	0.6	14.9	0.6	16.0		0.6	16.0		0.7	15.9		0.5	14.4		0.4	0.9906

The plots were cut in June 2011 and 2012 **(first cuts**; for second cuts of all but ME and NEL see Fig 3 and S1 Fig). Response parameters are biomass (g m^-2^), XF = crude fibre (% in dw), XP = crude protein (% in dw), DE = digestible energy (MJ kg^-1^ dw), ME = metabolisable energy (MJ kg^-1^ dw), NEL = net energy for lactation (MJ kg^-1^ dw), fructan (% in dw) and N-, P-, K-contents (mg g^-1^ dw); dw = dry weight. Values are means (*x*) + SE; n = 3. The right column indicates differences between moist and dry sites within years. Only P-content differs between treatment groups at the dry site in 2011 (different letters indicating significant differences at p<0.05).

**Table 3:** Forage quality parameters of differently treated meadow plots (control, +N, -25%, and +N/-25%) at the Elbe tributaries (oceanic site at the Sude and more continental site at the Havel River).

	Sude (oceanic site)								Havel (more continental site)								Significance
	control		+N		-25%		+N/-25%		control		+N		-25%		+N/-25%		between sites
	*x*	SE	*x*	SE	*x*	SE	*x*	SE	*x*	SE	*x*	SE	*x*	SE	*x*	SE	*p*
**Sep. 2009**																	
Biomass	197.9	11.0	200.2	13.8	192.6	10.2	217.5	15.8	211.6	16.4	211.0	9.7	190.4	12.8	199.0	13.5	1.0000
XF	30.4	0.4	28.7	0.5	29.1	0.4	29.2	0.5	27.1	0.7	26.9	0.6	28.2	0.6	27.1	0.6	0.0011
XP	10.7	0.4	10.9	0.3	10.3	0.5	10.9	0.4	10.2	0.6	11.1	0.5	10.4	0.5	10.3	0.4	0.9962
DE	8.0	0.1	8.4	0.1	8.3	0.2	8.3	0.1	8.9	0.2	8.9	0.2	8.7	0.2	8.9	0.2	<0.0001
ME	7.6	0.1	7.8	0.1	7.9	0.2	8.0	0.1	8.2	0.2	8.2	0.2	8.0	0.2	8.3	0.2	0.0302
NEL	4.3	0.1	4.5	0.1	4.5	0.1	4.6	0.1	4.7	0.1	4.7	0.1	4.6	0.1	4.8	0.1	0.0307
Fructan	0.4	0.2	0.7	0.3	1.3	0.6	0.9	0.7	1.7	0.4	1.4	0.4	2.9	0.5	2.7	0.3	0.0088
N	17.6	0.5	17.9	0.3	17.3	0.7	18.1	0.6	16.2	0.9	17.5	0.6	16.2	0.8	16.2	0.6	0.0855
P	1.8	0.2	1.7	0.1	1.9	0.2	1.9	0.3	2.9	0.2	2.7	0.1	2.8	0.2	2.9	0.2	0.0001
K	7.2	0.4	6.8	0.2	7.8	0.2	8.8	0.2	7.0	0.5	6.8	0.3	6.8	0.3	6.6	0.4	0.0546
**June 2010**																	*p*
Biomass	363.4	52.5	392.3	49.0	381.5	37.2	445.1	32.4	360.4	25.0	405.2	20.2	427.9	28.6	429.8	26.5	0.9689
XF	30.2	0.7	29.9	1.1	30.6	0.7	30.5	0.5	28.1	0.9	29.5	0.8	32.0	0.6	29.6	0.8	0.8640
XP	10.2	0.5	9.7	0.4	10.4	0.6	10.3	0.2	12.5	1.0	11.6	0.7	10.2	0.4	10.5	0.5	0.0381
DE	8.3	0.1	8.4	0.2	8.3	0.2	8.3	0.1	8.8	0.2	8.5	0.2	8.1	0.2	8.6	0.1	0.4694
ME	8.3	0.2	8.5	0.1	8.5	0.1	8.5	0.1	8.9	0.2	8.7	0.2	8.4	0.1	8.8	0.1	0.0684
NEL	4.8	0.1	4.9	0.1	4.9	0.1	4.9	0.1	5.2	0.1	5.0	0.1	4.9	0.1	5.1	0.1	0.0685
Fructan	3.0	0.8	3.8	0.9	3.2	0.7	2.6	0.5	2.6	0.8	2.6	0.6	3.4	0.6	3.2	0.8	0.9747
N	16.5	0.4	16.6	0.5	17.6	0.5	18.3	0.8	19.1	1.5	17.1	0.5	17.2	0.6	16.4	0.6	0.9812
P	2.0	0.1	2.0	0.1	2.3	0.1	2.1	0.1	3.3	0.2	2.9	0.1	3.0	0.2	3.1	0.2	0.0002
K	10.5	0.3	10.0	0.3	11.3	0.6	11.1	0.5	13.8	1.0	13.2	0.8	14.0	0.8	13.6	1.0	0.0002
**Sep. 2010**																	
Biomass	174.0	25.1	162.9	13.1	174.1	21.2	193.2	9.0	231.0	24.6	264.4	19.1	252.1	19.2	251.8	27.1	0.0001
XF	26.2	0.8	26.3	1.2	24.9	0.5	26.5	0.7	24.1	0.8	25.5	0.6	25.9	0.7	25.1	0.5	0.5913
XP	14.1	0.5	13.5	0.5	14.0	0.5	14.1	0.7	13.7	0.6	13.6	0.7	13.2	0.4	13.7	0.4	0.9088
DE	8.8	0.2	8.8	0.3	9.1	0.2	8.9	0.1	9.6	0.2	9.3	0.2	9.2	0.2	9.3	0.2	0.0027
ME	8.5	0.2	8.4	0.2	8.6	0.2	8.6	0.1	9.2	0.2	9.0	0.2	9.0	0.2	9.1	0.2	<0.0001
NEL	4.9	0.1	4.9	0.1	5.0	0.1	5.0	0.1	5.4	0.1	5.3	0.2	5.2	0.1	5.4	0.1	<0.0001
Fructan	0.5	0.5	1.1	0.5	0.2	0.2	0.6	0.5	1.2	0.3	1.7	0.7	2.3	1.0	1.1	0.5	0.1856
N	22.5	0.7	22.5	0.8	22.9	0.7	23.4	0.6	21.4	0.9	21.3	0.9	21.0	0.4	20.6	0.3	0.0029
P	2.3	0.1	2.3	0.1	2.7	0.2	2.4	0.2	3.7	0.2	3.5	0.3	3.5	0.2	3.4	0.3	0.0001
K	11.1	0.4	9.5	0.4	10.6	0.3	10.4	0.2	11.4	0.6	11.5	0.8	11.1	0.5	11.0	0.6	0.0781
**Overall yield 2010**	45.3	6.0	46.8	4.1	47.5	4.4	54.5	2.8	43.4	4.3	58.8	1.9	50.4	4.1	60.1	4.3	0.0046
**June 2011**																	
Biomass	194.8	26.7	227.2	35.1	199.8	19.0	204.1	29.1	383.4	33.0	340.4	32.7	372.2	21.6	372.5	19.6	0.0002
XF	27.5	1.1	27.5	1.2	26.8	1.2	27.4	1.0	25.6	0.6	24.8	0.8	26.9	0.7	25.8	0.7	0.0594
XP	11.7	0.4	12.0	0.5	11.5	0.3	12.6	0.7	11.6	0.7	11.9	0.6	10.9	0.3	11.1	0.6	0.4492
DE	8.7	0.2	8.7	0.2	8.9	0.2	8.7	0.2	9.1	0.1	9.3	0.2	8.8	0.1	9.1	0.1	0.0720
ME	8.6	0.2	8.6	0.2	8.7	0.2	8.6	0.2	9.1	0.1	9.2	0.1	8.9	0.1	9.1	0.1	0.0014
NEL	5.0	0.1	5.0	0.1	5.1	0.1	5.0	0.1	5.3	0.1	5.4	0.1	5.2	0.1	5.3	0.1	0.0011
Fructan	1.5	0.4	2.0	0.5	1.6	0.6	0.7	0.2	3.7	0.9	4.4	0.6	4.4	0.6	4.3	0.6	0.0002
N	19.8	0.7	20.5	0.5	21.5	0.6	20.9	0.4	19.1	1.1	18.3	0.6	17.3	0.4	18.1	0.6	0.0002
P	1.9	0.1	2.0	0.1	2.2	0.1	2.0	0.2	2.8	0.1	2.6	0.1	2.5	0.1	2.5	0.1	0.0002
K	10.7	0.4	9.4	0.6	10.1	0.7	9.8	0.6	11.7	0.5	12.0	0.7	11.6	0.7	11.4	0.6	0.0032
**Sep. 2011**																	
Biomass	245.7	13.1	289.1	31.6	270.5	29.8	269.3	17.9	343.3	20.9	354.4	18.7	326.3	21.0	345.2	13.0	0.0001
XF	26.4	1.0	27.7	0.7	25.9	0.9	27.9	0.9	24.2	0.8	25.4	0.5	26.0	0.3	26.1	0.6	0.0273
XP	12.9	0.7	13.4	0.9	12.9	0.5	13.9	0.7	12.7	0.6	12.5	0.3	12.2	0.7	12.5	0.8	0.3485
DE	8.4	0.2	8.2	0.2	8.5	0.1	8.2	0.2	9.0	0.1	8.7	0.1	8.7	0.1	8.7	0.1	0.0016
ME	8.2	0.1	8.0	0.1	8.1	0.1	8.0	0.2	8.7	0.1	8.5	0.1	8.5	0.1	8.5	0.1	0.0004
NEL	4.7	<0.1	4.5	0.1	4.7	0.1	4.6	0.1	5.1	0.1	5.0	<0.1	5.0	0.1	5.0	0.1	0.0003
Fructan	0.8	0.3	1.7	1.0	1.0	0.5	1.5	0.6	1.7	0.7	1.7	0.5	2.4	0.8	1.8	0.7	0.6503
N	21.2	0.5	22.2	0.8	21.2	0.6	22.8	0.6	19.5	0.6	19.2	0.3	18.8	0.5	19.0	0.7	0.0001
P	2.8	0.2	3.2	0.2	3.2	0.2	3.5	0.3	3.6	0.2	3.4	0.2	3.3	0.3	3.3	0.2	0.6205
K	8.6	0.5	7.7	0.2	8.1	0.5	8.3	0.3	11.5	0.7	11.3	0.4	11.4	0.6	11.2	0.6	<0.0001
**Overall yield 2011**	37.3	2.8	43.0	4.3	40.7	3.1	39.8	3.5	65.4	3.4	62.4	3.5	61.1	3.3	63.7	2.4	0.0002

The plots were cut in June 2010 and 2011 **(first cuts)** and in September 2009, 2010 and 2011 **(second cuts)**. Response parameters are biomass (g m^-2^), XF = crude fibre (% in dw), XP = crude protein (% in dw), DE = digestible energy (MJ kg^-1^ dw), ME = metabolisable energy (MJ kg^-1^ dw), NEL = net energy for lactation (MJ kg^-1^ dw), fructan (% in dw), N-, P-, K-contents (mg g^-1^ dw), and annual yield (GJ ha^-1^); dw = dry weight. Values are means (*x*) + SE. n = 7 for all groups. No differences were detected between treatments within sites and years, only between sites (right column).

On the Elbe sites, biomass samples were taken from 0.25 m^2^ subplots (three samples of biomass of which one was taken for the forage quality measurements and two were used for the element content measurements) in June 2010 and 2011 (first cuts) and September 2009, 2010 and 2011 (second cuts). At the Rhine sites, biomass samples were taken from 0.1 m^2^ subplots (12 samples which were ground together and then separated for the forage quality and element content measurements) in June 2011 and 2012 (first cuts) and September 2011 and 2012 (second cuts). In autumn 2011, not enough biomass could be sampled for the fructan analysis on the plots at the Rhine. Biomass samples of all sites were dried at 60°C for three days.

Crude protein, crude fibre, energy variables, and fructan were estimated using a NIRSystem 5000 (Foss GmbH Rellingen, Germany) and scanned between 1100 and 2500 nm. Annual yield (GJ ha^-1^) was calculated as the product of digestible energy (DE, GJ kg^-1^ dw) and dry biomass (kg dw ha^-1^). N contents of the Elbe samples were measured using a CN-Analyzer (vario MAX, elementar, Hanau, Germany) and of the Rhine samples using an Auto-Analyzer (AA 3, Bran & Lübbe, Norderstedt, Germany). K and P contents of the Elbe samples were measured with the ICP-OES technique (samples of 2009: Perkin Elmer ICP/OES, Perkin Elmer, Hamburg, Germany; samples of 2010/2011: iCAP^TM^ 6300 ICP-OES Analyzer, Thermo Scientific, Germany) after digestion of the samples in a Lab microwave (MLS Start 1500, Leutkirch, Germany). The Rhine samples were dry ash combusted and afterwards P contents were measured photometrically (Spectrophotometer, Zeiss, Jena, Germany) and K contents were measured using an Atomic Absorption Spectrometer (AAS-Varian 220 FS, Varian, Darmstadt, Germany).

### Data handling and statistical analyses

Due to the differences in study design at the Elbe and Rhine River, both data sets were analysed individually. The effects of the predictor variables on the response variables (annual yield, amount of biomass, crude protein, crude fibre, digestible energy, fructan, N-, P-, and K-content) were tested with repeated measures ANOVAs (with study year as the within subject factor). Except for annual yield, these analyses were done separately for the data from the first and the second cut. The fructan results of the Rhine samples (second cut) were analysed with a two-way ANOVA for the year 2012. In the analysis of the Elbe data set, the experimental predictor variables were precipitation reduction, N-addition, and site. In the analysis of the Rhine data set, the experimental predictor variables were the precipitation treatments (50% and 25% precipitation reduction, control with rainout-shelters, and control) and elevation above base flow (high, low). ANOVAs with significant results were followed by Tukey HSD-tests for comparisons between treatment groups. Basic requirements to conduct a parametric ANOVA such as normality and homoscedasticity were visually checked using diagnostic plots. All statistical tests were conducted using STATISTICA 10 (StatSoft Inc.).

## Results

### Rhine experiment

**Fig 2:**
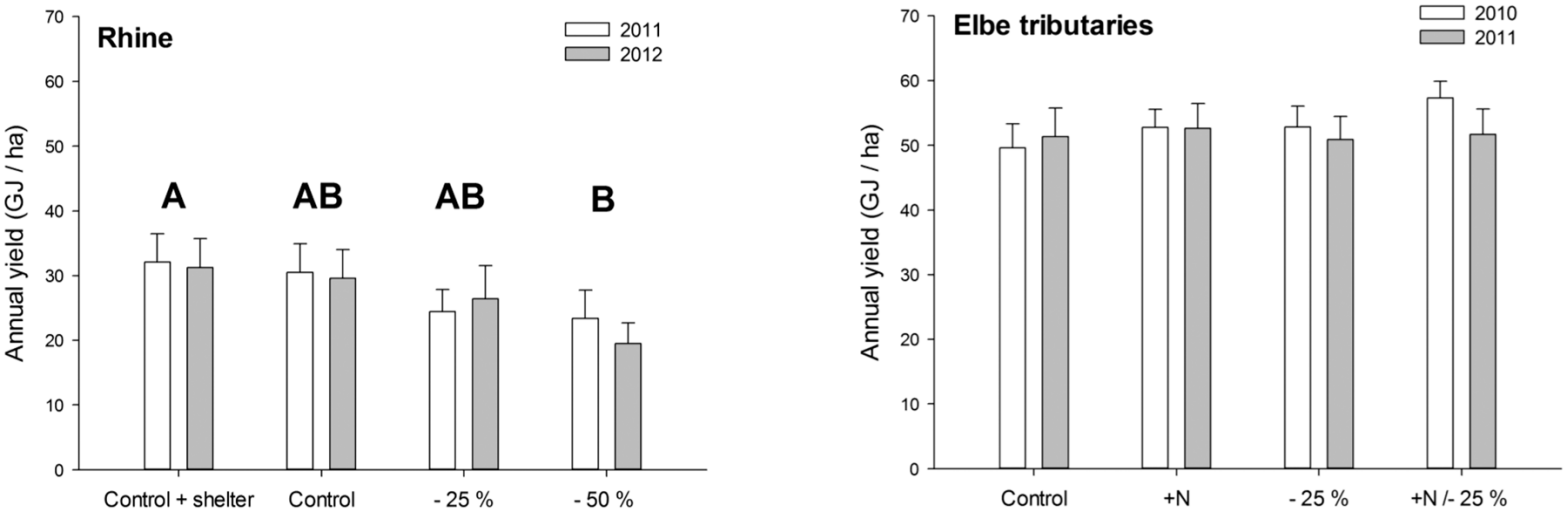
Annual yield (mean ± SE) from the meadows at the Rhine in the study years 2011 and 2012 (n = 6) and at the Elbe tributaries in 2010 and 2011 (n = 14). The study sites were pooled. Different letters indicate significant differences at p<0.05 across the study years.

The total annual yield significantly decreased by app. 30% under 50% precipitation reduction (21.5 ± 2.6 GJ ha^-1^) compared to the controls with rainout-shelters (31.7 ± 3.0 GJ ha^-1^; repeated measures ANOVA: *F*_3,16_ = 4.3, *p* = 0.0215; Tukey: *p* = 0.0248; Fig 2) and was higher on the lower (35.2 ± 1.8 GJ ha^-1^) compared to the higher site (19.0 ± 0.6 GJ ha^-1^; repeated measures ANOVA: *F*_1,16_ = 52.3, *p* < 0.0001; Tukey: *p* = 0.0002).

In the first cut, no main effects of experimental treatments on the amount of biomass (overall mean ± SE: 222.3 ± 13.1 g m^-2^) or on the forage quality variables crude fibre (35.5 ± 0.4% dw), crude protein (8.5 ± 0.3% dw), digestible energy (7.3 ± 0.1 MJ kg^-1^ dw), net energy for lactation (4.2 ± 0.1 MJ kg^-1^ dw), metabolisable energy (7.5 ± 0.1 MJ kg^-1^ dw), and the N- and K-contents (N: 12.7 ± 0.4 mg g^-1^ dw; K: 15.9 ± 0.2 mg g^-1^ dw) were detected. Only the fructan content significantly increased with 50% reduced precipitation (3.0 ± 0.4% dw) compared to the controls with rainout shelters (1.8 ± 0.4% dw; repeated measures ANOVA: *F*_3,16_ = 7.9, *p* = 0.0019; Tukey: *p* = 0.0023) and without rainout shelters (2.0 ± 0.4% dw; Tukey: *p* = 0.0110). Further, the P-content was significantly affected by precipitation reduction (repeated measures ANOVA: *F*_3,16_ = 4.2, *p* = 0.0221) and by an interaction between ‘precipitation reduction’ and ‘site’ (repeated measures ANOVA: *F*_3, 16_ = 4.7, *p* = 0.0151). The P-content of the biomass decreased at 50% precipitation reduction (2.9 ± 0.1 mg g^-1^ dw) compared to the controls without rainout-shelters (3.3 ± 0.2 mg g^-1^ dw; Tukey: *p* = 0.0195) and the P-content differed between differently treated groups at the higher site in 2012 (see Table 2).

**Fig 3:**
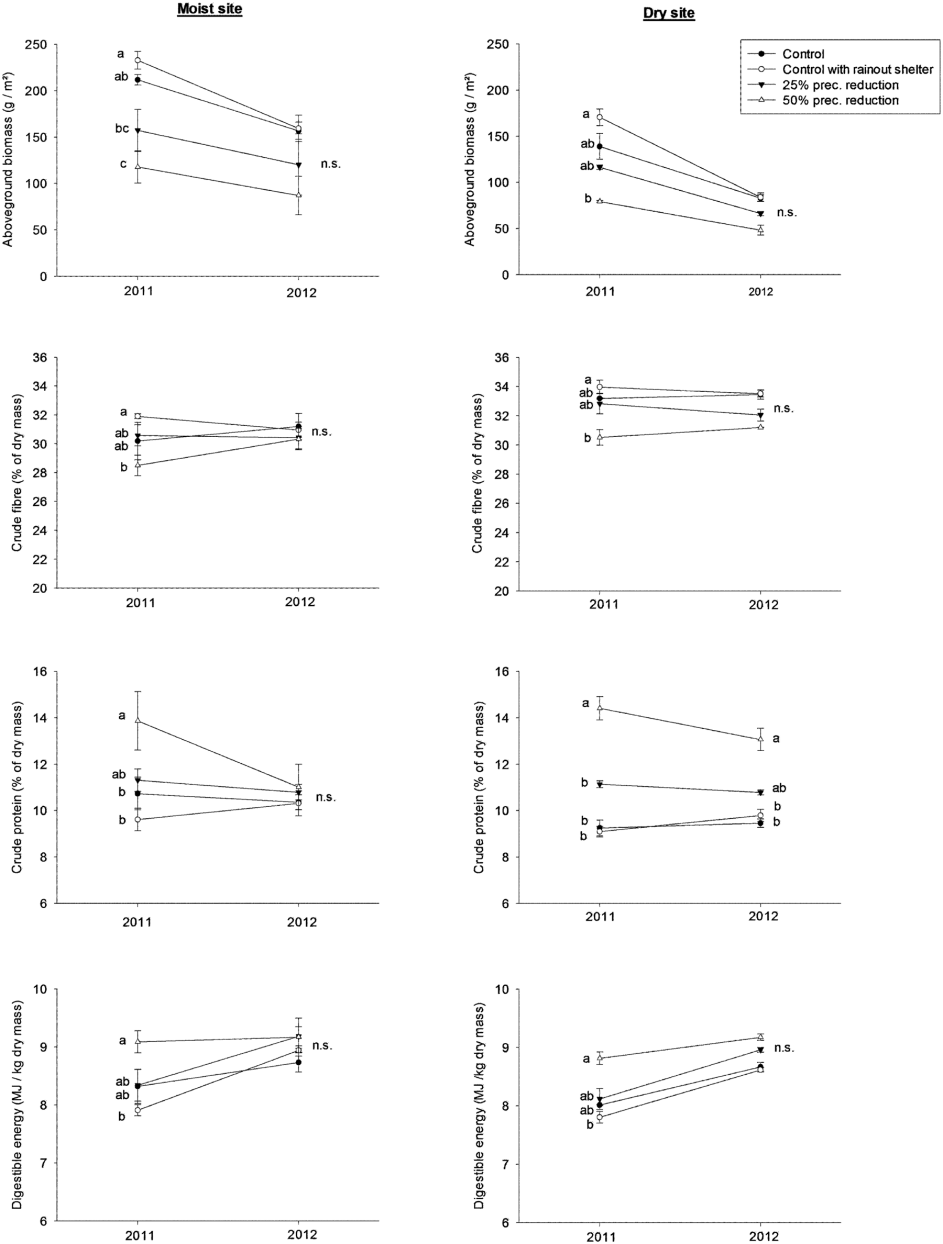
Responses of aboveground biomass and its content of crude fibre, crude protein and digestible energy to experimental treatments. Results refer to second cuts of Rhine sites of the years 2011 and 2012. Different letters indicate significant differences at p<0.05; (mean ± SE; n = 3).

In the second cut, all response variables (except for K-content and fructan) were affected by the precipitation treatments (Fig 3 and S1 Fig). The two differently elevated sites mainly reacted in the same way—an interaction between precipitation reduction and elevation was only detected for the response variable P-content (repeated measures ANOVA: *F*_3,16_ = 3.6, *p* = 0.0365). At both elevations, crude fibre and biomass decreased under precipitation reduction treatments while crude protein and digestible energy increased in 2011. Concerning the element contents, N-contents increased on the higher site by 32% between controls with rainout shelters and plots with 50% precipitation reduction, and P-contents increased by 30% between the same treatments on the lower site in 2011 (see S1 Fig). In 2012, the effects of precipitation treatments were generally less pronounced (see Fig 3 and S1 Fig).

### Elbe experiment

The total annual yield did not respond significantly to the precipitation reduction of 25% (repeated measures ANOVA, *F*_1,48_ = 0.4, *p* = 0.51, Fig 2).

In the first cut of the experimental grasslands in the Elbe region, no main effects of the experimental treatments were detected on any of the response variables (for mean values of the variables see Table 3). Significant differences occurred only between the two sites characterised by different climatic conditions (for *p*-values between sites within years see Table 3). and between the study years 2010 and 2011 (all *p* < 0.0001; except for fructan: *F*_1,48_ = 0.7, *p* = 0.3938).

In the second cut, the quantity and quality of the biomass did not respond to main effects of the experimental treatments. As in the first cut, significant differences were only detected between the two sites with differing climatic conditions (for *p*-values between sites within years see Table 3). Further, the differences between years (2009–2011) were significant for all response variables (repeated measures ANOVAs, all *p* < 0.0001) except for fructan (*F*_2,96_ = 2.5, *p* = 0.0878). Though not significant, the percentage of crude fibre tended to decrease under precipitation reduction at the oceanic site, and tended to increase under these conditions at the more continental site (repeated measures ANOVA, F_1,48_ = 2.9, *p* = 0.0941). Vice versa, the amount of digestible energy tended to increase under precipitation reduction at the oceanic site, while it tended to decrease under these conditions at the more continental site (repeated measures ANOVA, F_1,48_ = 3.2, *p* = 0.0797).

## Discussion

The productivity of the meadows in this study is in the same range as that of other flood meadows [47], but lower than that of non-flooded meadows [34] or fertilized semi-natural grasslands [36]. Generally, data on crude fibre, crude protein, energy content and fructan of European semi-natural grasslands are scarce in the literature. The few existing studies report values of these variables in the range of our study [17, 18, 47] or slightly higher NEL values in a calcareous grassland [48]. Our contents of N, P and K were very variable, but roughly in the same range [48, 49] or lower [36] compared to other studies on semi-natural grasslands. The hay quality of semi-natural floodplain meadows is low compared to agriculturally improved and intensively used sown hay meadows [36]. This is in accordance with Franke [47] who concluded that the hay originating from semi-natural meadows is especially suitable for leisure horses and young cattle or not lactating cows. For lactating cows, the energy content is too low [47] but can be incorporated into basic ration [50].

As our most important result, the annual yield decreased under precipitation reduction by 50% in the experiment at the Rhine River. However, the precipitation reduction of 25% did not affect the quantity and quality of hay from the meadows at all sites. Therefore, our findings indicate that climate change could affect the quantity and quality of hay in the future, when the reduction of precipitation is severe.

At the Rhine River, the responses of the meadow vegetation to the experimental treatments were more pronounced in the second than in the first cut. Most importantly, the amount of biomass and its content of crude fibre decreased under reduced precipitation in the second cut, while interestingly, the digestible energy and the contents of crude protein, N, and P increased in the biomass. Generally, an increase in hay quality in dry years was already reported by Opitz von Boberfeld [37], but the underlying processes still remain unclear. A possible explanation for the higher N-contents in the biomass from plots with reduced productivity could be the dilution effect, i.e. a decrease in N-concentrations through a proportionally higher accumulation of C than of N during growth of the aboveground biomass [26]. Secondly, the N-contents in the biomass might have been higher at the precipitation reduction treatment because of slower re-growth of the meadow plants after the first cut. Under dryer conditions, plant development is decelerated [25] and the aboveground biomass remains longer in an earlier phenological state, i.e. it consists mainly of leaves at harvest compared to plots with full precipitation, where plants already developed stems. The N concentration in leaves is greater than in stems and the N concentration of the whole herbage depends largely on the leaf/stem ratio [51]. A third explanation for the higher N- and protein contents under reduced precipitation might be a larger variability in soil moisture. Fluctuations in moisture content stimulate nutrient mineralisation [52]. Especially the extractable P pool was reported to increase upon soil re-wetting [53]. These fluctuations may be the reason why we detected higher P-contents in the biomass of the control plots in spring 2012 on the dry site while on the contrary we measured higher P-values under experimentally reduced precipitation in the plant foliage in autumn 2011 on the lower site. Drought stress also increased foliar N and P concentration of eastern cottonwood *Populus deltoides* [54] and in *Salix* spp. [55]. The observation that plants absorb nutrients at a higher rate than is required for their actual plant growth when another resource is limiting, is interpreted as advantageous as these absorbed nutrients can be readily incorporated in assimilates when the limiting resource is available again [56]. Further, higher concentrations of osmotic compounds (e.g. N containing amino-acids) decrease the water potential of tissues and thus helps the plants to take up water from the soil [57].

The N input of 35 kg ha^-1^ a^-1^ at the Elbe tributaries had no effect on the quantity and quality of the hay. Probably the experimental N input was negligible compared to inputs by flooding events, which are the main source of nutrients in floodplain ecosystems [58].

Besides of the weak treatment effects, our data demonstrate a high variability of the response variables between the study years at both rivers. This is probably due to the different weather conditions in the study years. Shortly before the second cuts, for example, the weather conditions were very dry in 2009, very wet in 2010, and intermediate in 2011 at the Elbe tributaries (see Fig 1a and 1b).

High variability of the response variables was also found between the experimental sites at both rivers. At the Elbe tributaries, it is striking that the responses of crude fibre and energy content were completely different between the two sites. A possible explanation for this pattern could be that the percentage of dicots differed between the sites. The oceanic site (with the tendency of reduced crude fibre and higher energy at reduced precipitation in the second cut) showed higher abundances of grasses compared to the more continental site, which tended to develop reduced energy and higher crude fibre in the second cut hay at reduced precipitation (see S2 Fig). The grasses mostly reproduce (i.e. set seeds) before the first cut in floodplain grasslands and grow leaves until the second cut. Since the N concentration (and energy content) is larger in leaves than in stems [51], leaves contain less crude fibre. In the more continental site, the dicots might have been in the process of reproduction at the second cut, in which the reduced precipitation might have had a ‘slow down’ effect on the reproduction process.

Overall, the meadows at the Rhine were more responsive to the experimental treatments than the Elbe meadows. This may, on the one hand, be due the lack of a 50% reduction treatment in the Elbe experiment. Therefore, the measured responses were limited and the effects at the Elbe River may be underestimated. On the other hand, the ‘new’ meadows of the Rhine floodplain were less species rich compared to the ‘old’ meadows at the Elbe tributaries. Species richness might have buffered possible effects of reduced precipitation on the response variables: in species-rich stands, some species may be facilitated through the treatments, compensating reduced growth of other species and thus increasing the reliability of grassland productivity under variable conditions [59, 60, 61]. Thus, the role of meadow age and species richness in the drought resilience of grasslands requires further research.

## Conclusions

The annual yield decreased under precipitation reduction of 50% in the experiment at the Rhine River. Therefore, we conclude that the yield of floodplain meadows may become less reliable in future. Nevertheless, the effects of the two precipitation reduction experiments on forage quality and the amount of biomass were, overall, rather small. This finding fits with other studies reporting weak or no effects of drought events on grassland productivity [32, 34, 62, 63]. The first cut was not affected by precipitation reduction (except for fructan at the Rhine sites) in the floodplain meadows at both rivers. As the first cut is usually of higher quantity and quality than the second cut and, therefore, economically more important for agricultural purposes, at least the use of the first cut hay may be still possible under climate change. In case of our studied floodplain meadows, soil moisture was probably sufficient for plant growth from winter/spring until the first cut in June. It has to be considered that the groundwater levels of floodplains correspond to the water level of the associated river. Consequently, whether the productivity of the floodplain meadows will be affected in future will largely depend on whether the river discharges will decrease during winter and spring in future. Finally, as multiple factors may simultaneously change in the course of climate change, additive effects of reduced summer precipitation, higher temperatures and increased CO_2_ concentrations, and possibly lowered groundwater tables are likely to influence productivity of floodplain meadows.

## Supporting Information

S1 Fig Responses of the content of fructan, N, P, and K to experimental treatments. Results refer to second cuts of Rhine-sites of the years 2011 and 2012. Different letters indicate significant differences at p<0.05; (Mean ± SE; n = 3).
(TIF)

S2 Fig The abundance of the functional groups ‘grasses’ and ‘herbs’ at the time of cutting in June and September 2011 at the experimental sites at the Elbe tributaries Sude and Havel. (TIF)

## References

[1] Billeter R, Liira J, Bailey D, Bugter R, Arens P, Augenstein I, et al Indicators for biodiversity in agricultural landscapes: a pan-European study: Biodiversity in European Agro-ecosystems. J Appl Ecol. 2008;45: 141–150. 10.1111/j.1365-2664.2007.01393.x

[2] Liira J, Schmidt T, Aavik T, Arens P, Augenstein I, Bailey D, et al Plant functional group composition and large-scale species richness in European agricultural landscapes. J Veg Sci. 2008;19: 3–14. 10.3170/2007-8-18308

[3] Hendrickx F, Maelfait J-P, Van Wingerden W, Schweiger O, Speelmans M, Aviron S, et al How landscape structure, land-use intensity and habitat diversity affect components of total arthropod diversity in agricultural landscapes: Agricultural factors and arthropod biodiversity. J Appl Ecol. 2007;44: 340–351. 10.1111/j.1365-2664.2006.01270.x

[4] Seffer J, Janák M, Sefferová Stanová V. Management models for habitats in Natura 2000 sites—6440 Alluvial meadows of river valleys of the *Cnidion dubii*—Directive 92/43/EEC on the conservation of natural habitats and of wild fauna and flora. European Commission; 2008. Report No.: 17/24.

[5] Donath TW, Hölzel N, Otte A. The impact of site conditions and seed dispersal on restoration success in alluvial meadows. Appl Veg Sci. 2003;6: 13–22.

[6] Toogood S, Joyce C, Waite S. Response of floodplain grassland plant communities to altered water regimes. Plant Ecol. 2008;197: 285–298.

[7] Burkart M. River corridor plants (Stromtalpflanzen) in Central European lowland: a review of a poorly understood plant distribution pattern. Glob Ecol Biogeogr. 2001;10: 449–468.

[8] Hölzel N, Otte A. The impact of flooding regime on the soil seed bank of flood-meadows. J Veg Sci. 2001;12: 209–218.

[9] Tockner K, Stanford JA. Riverine flood plains: present state and future trends. Environ Conserv. 2002;29: 308–330.

[10] Brunotte E, Dister E, Günther-Diringer D, Koenzen U, Mehl D, Amberger P, et al Flussauen in Deutschland—Erfassung und Bewertung des Auenzustandes. Naturschutz und Biologische Vielfalt. 2009; 87: 3–139.

[11] Wesche K, Krause B, Culmsee H, Leuschner C. Fifty years of change in Central European grassland vegetation: Large losses in species richness and animal-pollinated plants. Biol Conserv. 2012;150: 76–85.

[12] Jensen K, Schrautzer J. Consequences of abandonment for a regional fen flora and mechanisms of successional change. Appl Veg Sci. 1999;2: 79–88. 10.2307/1478884

[13] Jensen K, Trepel M, Merritt D, Rosenthal G. Restoration ecology of river valleys. Basic Appl Ecol. 2006;7: 383–387.

[14] Donath TW, Bissels S, Hölzel N, Otte A. Large scale application of diaspore transfer with plant material in restoration practice—Impact of seed and microsite limitation. Biol Conserv. 2007;138: 224–234. 10.1016/j.biocon.2007.04.020

[15] Schmiede R, Otte A, Donath TW. Enhancing plant biodiversity in species-poor grassland through plant material transfer—the impact of sward disturbance. Appl Veg Sci. 2012;15: 290–298.

[16] Isselstein J, Jeangros B, Pavlu V. Agronomic aspects of biodiversity targeted management of temperate grasslands in Europe—A review. Agron Res. 2005;3: 139–151.

[17] Donath TW, Hölzel N, Bissels S, Otte A. Perspectives for incorporating biomass from non-intensively managed temperate flood-meadows into farming systems. Agric Ecosyst Environ. 2004;104: 439–451. 10.1016/j.agee.2004.01.039

[18] Donath TW, Schmiede R, Otte A. Alluvial grasslands along the northern upper Rhine—nature conservation value vs. agricultural value under non-intensive management. Agric Ecosyst Environ. 2015;200: 102–109. 10.1016/j.agee.2014.11.004

[19] Huxman TE, Smith MD, Fay PA, Knapp AK, Shaw MR, Loik ME, et al Convergence across biomes to a common rain-use efficiency. Nature. 2004;429: 651–654. 10.1038/nature02561 15190350

[20] IPCC. Climate Change 2007: The Physical Science Basis Contribution of Working Group I to the Fourth Assessment Report of the Intergovernmental Panel on Climate Change. Cambridge & New York; 2007 p. 996.

[21] Jacob D, Göttel H, Kotlarski S, Lorenz P, Sieck K. Klimaauswirkungen und Anpassung in Deutschland—Phase 1: Erstellung regionaler Klimaszenarien für Deutschland. Dessau-Roßlau: UBA—Umwelt Bundesamt 2008. Report No.: 08/11.

[22] Görgen K, Beersma J, Brahmer G, Buiteveld H, Carambia M, de Keizer O, et al Assessment of climate change impacts on discharge in the Rhine Basin: Results of the RheinBlick2050 Project. Lelystad; 2010.

[23] Conradt T, Koch H, Hattermann FF, Wechsung F. Spatially differentiated management-revised discharge scenarios for an integrated analysis of multi-realisation climate and land use scenarios for the Elbe River basin. Reg Environ Change. 2012;12: 633–648.

[24] Jensen K, Reisdorff C, Pfeiffer EM, Oheimb G von, Schmidt K, Schmidt S, et al Klimabedingte Änderungen in terrestrischen und semi-terrestrischen Ökosystemen In: Storch H von, Claussen M (editors) Klimabericht für die Metropolregion Hamburg. Berlin: Springer; 2011 pp. 143–176.

[25] Hsiao TC, Acevedo E. Plant responses to water deficits, water-use efficiency, and drought resistance. Agric Meteorol. 1974;14: 59–84. 10.1016/0002-1571(74)90011-9

[26] Hejcman M, Szaková J, Schellberg J, Tlustoš P. The Rengen Grassland Experiment: relationship between soil and biomass chemical properties, amount of elements applied, and their uptake. Plant Soil. 2010;333: 163–179. 10.1007/s11104-010-0332-3

[27] Pan JJ, Widner B, Ammerman D, Drenovsky RE. Plant community and tissue chemistry responses to fertilizer and litter nutrient manipulations in a temperate grassland. Plant Ecol. 2009;206: 139–150. 10.1007/s11258-009-9630-3

[28] Brum OB, López S, García R, Andrés S, Calleja A. Influence of harvest season, cutting frequency and nitrogen fertilization of mountain meadows on yield, floristic composition and protein content of herbage. Rev Bras Zootec. 2009;38: 596–604. 10.1590/S1516-35982009000400002

[29] Yahdjian L, Sala O, Austin AT. Differential controls of water input on litter decomposition and nitrogen dynamics in the Patagonian steppe. Ecosystems. 2006;9: 128–141.

[30] Fay PA, Carlisle JD, Knapp AK, Blair JM, Collins SL. Altering Rainfall Timing and Quantity in a Mesic Grassland Ecosystem: Design and Performance of Rainfall Manipulation Shelters. Ecosystems. 2000;3: 308–319. 10.1007/s100210000028

[31] Grime JP, Brown VK, Thompson K, Masters GJ, Hillier SH, Clarke IP, et al The response of two contrasting limestone grasslands to simulated climate change. Science. 2000;289: 762–765. 10.1126/science.289.5480.762 10926535

[32] Bloor JMG, Pichon P, Falcimagne R, Leadley P, Soussana J-F. Effects of warming, summer drought, and CO_2_ enrichment on aboveground biomass production, flowering phenology, and community structure in an upland grassland ecosystem. Ecosystems. 2010;13: 888–900. 10.1007/s10021-010-9363-0

[33] Bütof A, von Riedmatten LR, Dormann CF, Scherer-Lorenzen M, Welk E, Bruelheide H. The responses of grassland plants to experimentally simulated climate change depend on land use and region. Glob Change Biol. 2012;18: 127–137.

[34] Walter J, Grant K, Beierkuhnlein C, Kreyling J, Weber M, Jentsch A. Increased rainfall variability reduces biomass and forage quality of temperate grassland largely independent of mowing frequency. Agric Ecosyst Environ. 2012;148: 1–10.

[35] Kleinebecker T, Weber H, Hölzel N. Effects of grazing on seasonal variation of aboveground biomass quality in calcareous grasslands. Plant Ecol. 2011;212: 1563–1576.

[36] Tallowin JRB, Jefferson RG. Hay production from lowland semi-natural grasslands: a review of implications for ruminant livestock systems. Grass Forage Sci. 1999;54: 99–115. 10.1046/j.1365-2494.1999.00171.x

[37] Opitz von Boberfeld W. Grünlandlehre: biologische und ökologische Grundlagen. Stuttgart: E. Ulmer; 1994.

[38] Leyer I. Predicting plant species’ responses to river regulation: the role of water level fluctuations. J Appl Ecol. 2005;42: 239–250.

[39] Bissels S, Donath TW, Hölzel N, Otte A. Ephemeral wetland vegetation in irregularly flooded arable fields along the northern Upper Rhine: the importance of persistent seedbanks. Phytocoenologia. 2005;35: 469–488. 10.1127/0340-269x/2005/0035-0469

[40] Burmeier S, Eckstein RL, Otte A, Donath TW. Desiccation cracks act as natural seed traps in flood-meadow systems. Plant Soil. 2010;333: 351–364.

[41] Böger K. Grünlandvegetation im Hessischen Ried—Pflanzensoziologische Verhältnisse und Naturschutzkonzeption. Bot Naturschutz Hess. 1991;3.

[42] Bissels S, Hölzel N, Donath TW, Otte A. Evaluation of restoration success in alluvial grasslands under contrasting flooding regimes. Biol Conserv. 2004;118: 641–650. 10.1016/j.biocon.2003.10.013

[43] Wisskirchen R, Haeupler H. Standardliste der Farn- und Blütenpflanzen Deutschlands mit Chromosomenatlas von Focke Albers. 1st ed Stuttgart (Hohenheim): Eugen Ulmer GmbH & Co; 1998.

[44] Yahdjian L, Sala OE. A rainout shelter design for intercepting different amounts of rainfall. Oecologia. 2002;133: 95–101. 28547315 10.1007/s00442-002-1024-3

[45] Longland AC, Dhanoa MS, Harris PA. Comparison of a colorimetric and a high-performance liquid chromatography method for the determination of fructan in pasture grasses for horses. J Sci Food Agric. 2012;92: 1878–1885. 10.1002/jsfa.5555 22297902

[46] Bailey SR, Bamford NJ. Metabolic responses of horses and ponies to high and low glycaemic feeds: implications for laminitis. Anim Prod Sci. 2013;53: 1182–1187.

[47] Franke C. Grünland an der unteren Mittelelbe—Vegetationsökologie und landwirtschaftliche Nutzbarkeit. Berlin: Gebrüder Borntraeger; 2003.

[48] Kleinebecker T, Weber H, Hölzel N. Effects of grazing on seasonal variation of aboveground biomass quality in calcareous grasslands. Plant Ecol. 2011;212: 1563–1576. 10.1007/s11258-011-9931-1

[49] Olde Venterink H, Vermaat JE, Pronk M, Wiegman F, van der Lee GEM, van den Hoorn MW, et al Importance of sediment deposition and denitrification for nutrient retention in floodplain wetlands. Appl Veg Sci. 2006;9: 163–174.

[50] NRC—National Research Council. Nutrient requirements of dairy cattle. Washington, DC: National Academy Press; 2001.

[51] Duru M, Cruz P, Ansquer P, Navas ML. Standing herbage mass: An integrated indicator of management practices for examining how fertility and defoliation regime shape the functional structure of species-rich grasslands. Ecol Indic. 2014;36: 152–159. 10.1016/j.ecolind.2013.07.015

[52] Bloor JMG, Bardgett RD. Stability of above-ground and below-ground processes to extreme drought in model grassland ecosystems: Interactions with plant species diversity and soil nitrogen availability. Perspect Plant Ecol Evol Syst. 2012;14: 193–204. 10.1016/j.ppees.2011.12.001

[53] Olde Venterink H, Davidsson T, Kiehl K, Leonardson L. Impact of drying and re-wetting on N, P and K dynamics in a wetland soil. Plant Soil. 2002;243: 119–130.

[54] Broadfoot WM, Farmer RE. Genotype and moisture supply influence nutrient content of eastern cottonwood foliage. For Sci. 1969;15: 46–48.

[55] Weih M, Bonosi L, Ghelardini L, Rönnberg-Wästljung AC. Optimizing nitrogen economy under drought: increased leaf nitrogen is an acclimation to water stress in willow (*Salix* spp.). Ann Bot. 2011;108: 1347–1353. 10.1093/aob/mcr227 21896572 PMC3197455

[56] Chapin FS. The mineral nutrition of wild plants. Annu Rev Ecol Syst. 1980;11: 233–260. 10.1146/annurev.es.11.110180.001313

[57] Morgan JM. Osmoregulation and water stress in higher plants. Annu Rev Plant Physiol. 1984;35: 299–319. 10.1146/annurev.pp.35.060184.001503

[58] Beltman B, Willems JH, Güsewell S. Flood events overrule fertiliser effects on biomass production and species richness in riverine grasslands. J Veg Sci. 2007;18: 625–634.

[59] Chapin F, Zavaleta E, Eviner V, Naylor R, Vitousek P, Reynolds H, et al Consequences of changing biodiversity. Nature. 2000;405: 234–242. 10821284 10.1038/35012241

[60] Tilman D, Downing JA. Biodiversity and stability in grasslands. Nature. 1994;367: 363–365. 10.1038/367363a0

[61] Kleinebecker T, Hölzel N, Prati D, Schmitt B, Fischer M, Klaus VH. Evidence from the real world: ^15^N natural abundances reveal enhanced nitrogen use at high plant diversity in Central European grasslands. Jones R, editor. J Ecol. 2014;102: 456–465. 10.1111/1365-2745.12202

[62] Kreyling J, Wenigmann M, Beierkuhnlein C, Jentsch A. Effects of extreme weather events on plant productivity and tissue die-back are modified by community composition. Ecosystems. 2008;11: 752–763. 10.1007/s10021-008-9157-9

[63] Jentsch A, Kreyling J, Elmer M, Gellesch E, Glaser B, Grant K, et al Climate extremes initiate ecosystem-regulating functions while maintaining productivity. J Ecol. 2011;99: 689–702.

